# Cardiac Magnetic Resonance Analysis of Mitral Annular Dynamics after Mitral Valve Repair

**DOI:** 10.6061/clinics/2020/e2428

**Published:** 2020-11-10

**Authors:** Ahmad A. Abdouni, Carlos M.A. Brandão, Carlos E. Rochitte, Pablo M.A. Pomerantzeff, Elinthon T. Veronese, Ariane B. Pacheco, Antonio S. Santis, Flávio Tarasoutchi, Fábio B. Jatene

**Affiliations:** Instituto do Coracao (InCor), Hospital das Cinicas (HCFMUSP), Faculdade de Medicina, Universidade de Sao Paulo, Sao Paulo, SP, BR

**Keywords:** Cardiac Magnetic Resonance, Mitral Valve Insufficiency, Mitral Valve Repair

## Abstract

**OBJECTIVES:**

The aim of this study was to analyze mitral annulus (MA) dynamics using cardiac magnetic resonance (CMR) in patients with degenerative mitral insufficiency who underwent mitral valve repair (MVR).

**METHODS:**

Mitral valve imaging was performed by CMR in twenty-nine patients with degenerative mitral insufficiency who underwent MVR between July 2014 and August 2016, with quadrangular resection of the posterior leaflet without ring annuloplasty. They were prospectively followed up from the preoperative period up to 2 years postoperatively.

**RESULTS:**

We observed a significant reduction in all measurements of the MA after surgery. The mean systolic circumference of the MA was reduced from 13.28±1.95 cm to 11.50±1.59 cm, and the diastolic circumference was reduced from 12.51±2.01 cm to 10.66±2.09 cm in the immediate postoperative period, measures that remained stable 2 years after MVR (*p*<0.001). The mean maximum area of the MA was significantly reduced from 14.34±4.03 to 10.45±3.17 cm^2^ when comparing the immediate postoperative period and the 2 year follow-up (*p*<0.001). The same occurred with the mean minimum area of the MA, which was reduced from 12.53±3.68 cm^2^ to 9.23±2.84 cm^2^ in the same period, and this reduction was greater in the antero-posterior diameter than in the mid-lateral diameter. The mobility of the MA was preserved after surgery, ranging between 19.6% and 25.7% at 2-year follow-up.

**CONCLUSION:**

We observed a significant reduction in the MA size after MVR, with preservation of the MA mobility at the 2-year follow-up.

## INTRODUCTION

Mitral valve repair (MVR) is the treatment of choice for degenerative mitral regurgitation, presenting lower rates of thromboembolism and endocarditis, reduced need for anticoagulation, excellent survival and durability in long-term follow-up, and better left ventricular function, when compared with mitral valve (MV) replacement (1).

Recent studies have demonstrated that cardiac magnetic resonance (CMR) can accurately demonstrate variations in measurements and shape of the mitral annulus (MA) during the cardiac cycle (2,3).

There is little data in the literature regarding the remodeling and dynamics of the mitral annulus during the postoperative period of MVR. The present study describes aspects of the morphology and functioning of the MA over the course of a 2-year postoperative period, in a population with degenerative mitral regurgitation, with the use of CMR imaging to evaluate dimensions and mobility of the MA after MVR.

## MATERIAL AND METHODS

The present study was conducted at the Heart Institute of the University of São Paulo Medical School. The study protocol was approved by the Institutional Ethics Committee, and all patients provided written informed consent prior to the surgery.

Patients with multiple valvopathies, previous cardiac surgery, coronary artery disease, or those undergoing emergency surgery were excluded. From July 2014 to August 2016, 29 patients underwent MVR with the “Double Teflon” technique, which consists of the quadrangular resection of the prolapsed segment of the posterior leaflet, followed by annulus plication of the correspondent segment, reinforced with &quot;pledget&quot; stitches over the Teflon patch (4). All patients were studied by CMR through a specific protocol in the preoperative period, immediate postoperative period, and after 6 months, 1 year, and 2 years of follow-up. All procedures were performed by the same surgical team, and all examinations were also monitored by the same medical team.

**Figure 1:**
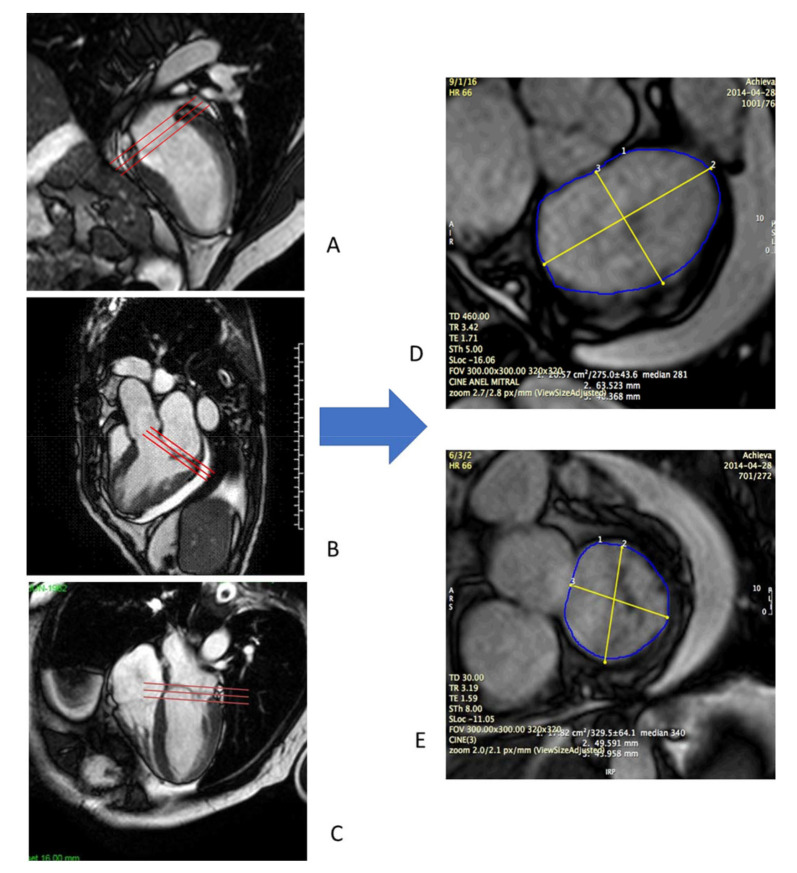
**Acquisition of mitral valve images by CMR.** We used three images for visualization of the mitral valve on the long axis, with images in two chambers (A), 3 chambers (B) and four chambers (C). Mitral valve insertion points were visualized in all of them and used as parameters to obtain an adequate alignment and to derive the images on the short axis of the mitral valve in systole (D) and diastole (E).

**Figure 2:**
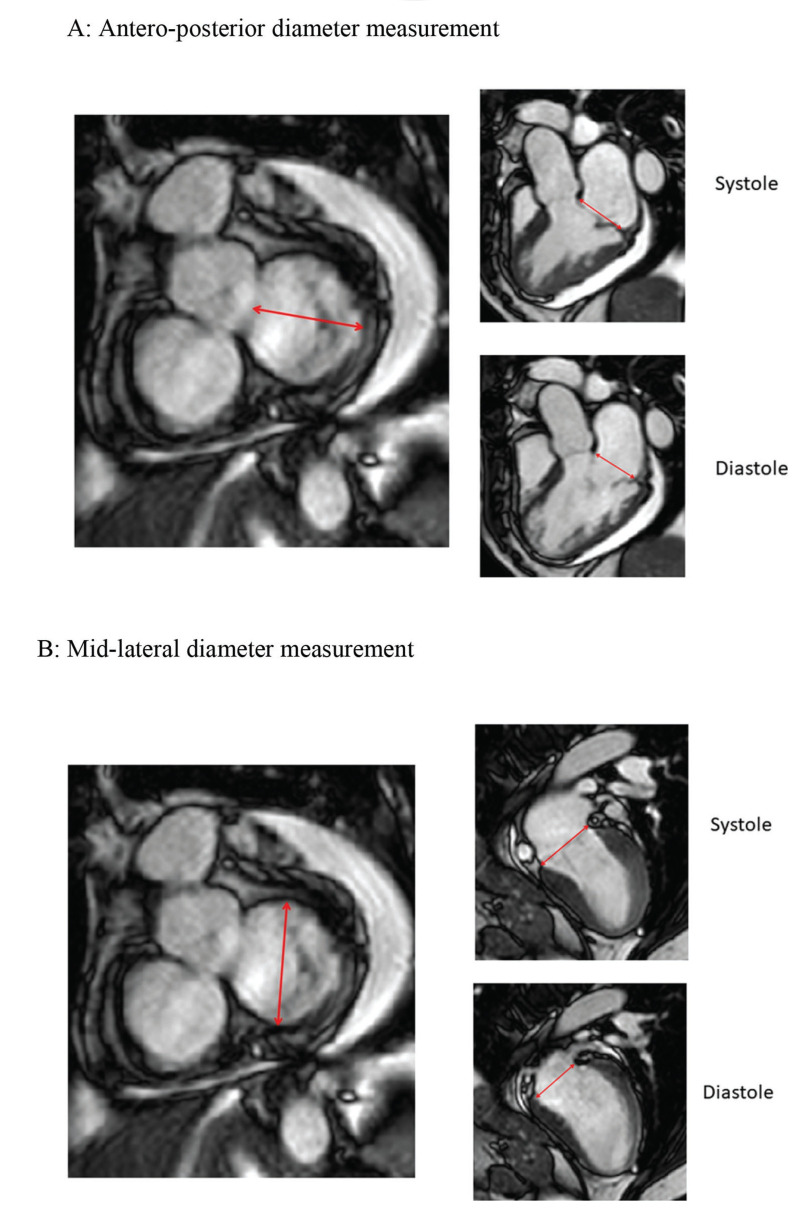
**Simultaneous imaging of the short and long axis grant for measurements of annular diameters.** A: Measurement of the antero-posterior diameter of the mitral annulus in the short axis derives an image in three chambers in the long axis, where the mitral valve insertion limits and insertion points are adjusted and checked. B: Measurement of mid-lateral diameter derives two-chamber images on long axis.

CMR studies were performed using a clinical 1.5 T scanner (Philips Achieva-Philips HealthCare, Best, Netherlands) using a phased-array receiver coil during breath-hold and electrocardiographic trigger. CMR images were acquired in multiple short-axis, long-axis-, and 3-chamber views in the standard format. Additional images were obtained specifically for better visualization of the MV annulus. We used 3 images for visualization of the MV on the long-axis view, with images of two chambers, three chambers, and four chambers. Using the insertion points of the MV as references in these three projections, we obtained an adequate alignment to generate the short-axis images of the MV annulus (Figure 1). We measured the antero-posterior (AP) and mid-lateral (ML) diameters of the MA as well as the circumference and the area of the MV annulus (Figure 2). All these measurements were obtained over four phases of the cardiac cycle: late diastole (D), early systole (S1), mid systole (S2), and late systole (S3), in order to assess the MA contractility.

Baseline characteristics and clinical outcomes are presented as absolute and relative values (percentage) for categorical values and mean and standard deviation for continuous measurements. The CMR continuous variables were compared preoperatively, and postoperatively at 30 days, 6 months, 1 year, and 2 years, using analysis of variance test with repeated measures. The software used for the analysis was SPSS 21.0 for Windows (5). The level of significance used for the tests was *p*<0.05

The mean age was 63.3 years (range, 40-81 years), and 17 patients were male (58.6%). The mean estimated surgical risk by STS Score was 1.13% (range, 0.35-4.9). Patient characteristics are presented in Table 1.

The mean cardiopulmonary bypass time was 66 minutes (range, 50-79 minutes), and the mean aortic cross-clamp time was 47 minutes (range, 36-58 minutes). The associated technique of MVR, the chordal shortening of the anterior leaflet, was used in five patients (17.2%).

Regarding the operative mitral anatomic findings, there was isolated prolapse of the P2 segment of the posterior leaflet in 19 patients (65.5%), isolated prolapse of P3 in 2 patients (6.9%), and one patient (3.5%) had isolated P1 prolapse. Seven patients (24.1%) had prolapse of two segments.

## RESULTS

There was one operative death (3.4%) secondary to acute cholecystitis and peritonitis. The remaining 28 patients were clinically reassessed at 6 months, 1 year, and 2 years postoperatively, when the follow-up CMR analysis was performed.

In the late clinical follow-up, two patients presented with embolic events (one stroke and one myocardial infarction), both successfully treated with anticoagulation. One patient presented with complete atrioventricular block 18 months after surgery and underwent pacemaker implantation. There were two late deaths (7.1%), both from non-cardiac causes. At the 2-year follow-up, 75% of the patients were in functional class I and 25% were in functional class II (NYHA).

We observed a significant decrease in MA circumference after surgery, the mean systolic and diastolic circumference significantly decreased to 11.5 cm and 10.66 cm, respectively and, at the 2-year follow-up, the MA circumference was 11.60 cm and 10.76 cm in systole and diastole, respectively (*p*<0.001). In the analysis of the MA area, a significant reduction in the mean minimum and mean maximum area of the MA was observed after MVR, remaining after 2 years of follow-up (*p*<0.001). We observed a decrease in both AP and ML diameters in all periods, and these remained stable during the 2 years of follow-up. Reduction was greater in the AP diameter, ranging from 18.04 to 21.76%. In the ML diameter, the reduction ranged from 7.8% to 9.9% (Table 2).

MA mobility was assessed by the variability of MV area over the cardiac cycle. There was no statistical difference between these measurements (*p*=0.572), as presented in Table 3.

## DISCUSSION

There are several surgical techniques to correct mitral insufficiency, including interventions on the chordae tendineae, on the leaflets, on the papillary muscles, and on the MA (6). Evidence has emerged regarding the benefits of preserving the mitral annulus physiology avoids changes in intraventricular blood flow and subsequently avoids left ventricular dysfunction. In addition, the use of prosthetic rings may interfere with the physiologic mobility of the MA, changing the saddle shape, making the annulus flatter and fastening it (7). In an experimental study using CMR for flow analysis, Witschey et al. demonstrated that prosthetic rings modify the intraventricular blood flow, which can lead to left ventricular dysfunction (8). Komoda et al. demonstrated that the maintenance of MA mobility is closely related to the preservation of ventricular function. In patients who underwent annuloplasty without the use of a prosthetic ring, the MA dynamics and contraction of the base of the heart were similar to those of normal individuals (9). There is also evidence that the displacement and contractility of the atrioventricular plane can be responsible for approximately 60% of the systolic volume in adults (10).

Existing published literature shows that during MVR in degenerative disease, in cases of isolated prolapse of the posterior leaflet, the “Double Teflon” technique is sufficient to repair the MV, with good long-term clinical outcomes (11). Patients with single segment prolapse of the posterior leaflet represented 75.8% of our series, and isolated P2 prolapse was the most common defect observed, accounting for 65.5% of our sample. In a review carried out by Fundarò et al., 13 publications of mitral annuloplasty without prosthetic rings were analyzed, and they observed good outcomes, reproducibility and cost efficiency (12). In a recent publication, Garatti et al. compared two techniques of MVR: posterior annuloplasty with a flexible ring and double suture in the posterior annulus. Both techniques were associated with similar mortality, clinical outcomes, recurrence of regurgitation, and readmission rates in 11 years of follow-up (13).

In our series, we observed a significant reduction in MA circumference after MVR. At the 2 year follow-up, the MA circumference was 11.60 cm in systole and 10.76 cm in diastole, with a reduction of 12.65% and 13.98%, respectively, compared to preoperative measurements (*p*<0.001). Similar results were observed for the MA area. In the preoperative period, the mean area of the annulus ranged from 12.6 cm^2^ to 14.3 cm^2^ over the cardiac cycle, which demonstrates greater measurements compared to the normal values described in the literature (2,14,15). In the immediate postoperative period, there was a significant reduction of MV area in relation to the preoperative period (*p*<0.001), and this reduction remained significant for up to 2 years of follow-up, when the area varied from 9.23 cm^2^ to 10.45 cm^2^, demonstrating the stability of MV repair in our follow-up. This reduction was significant in both the AP and ML diameters (*p*<0.001) in the immediate and late postoperative period; the reduction was more evident in the AP diameter, which was 19-21% smaller after 2 years of follow-up than in the preoperative period. It is important to emphasize the stability of the MA measurements after MVR in all analyzed parameters, using this technique of MVR.

An important finding in the follow-up period was the maintenance of mitral annular dynamics. Despite the reduction in area, the mobility of the MA during the cardiac cycle remained at the same degree observed in the preoperative period. In the preoperative period, the mean variation in the MA during the cardiac cycle was 23.3%, similar to the measures observed at the two year follow-up (*p*=0.572), demonstrating that the MA dynamics were preserved with this surgical technique. The annulus dynamics and their morphological changes have recently become a component in advanced cardiac function analysis, as MV motion can indicate regional and global motion (16). Previous studies have already used 2D and 3D echocardiograms to analyze the behavior of the MA, and recent studies have demonstrated that CMR can accurately demonstrate variations in the measurements and shape of the MA during the cardiac cycle. Some authors have demonstrated that the MA reaches its greatest circumference and area at the end of systole, when the annulus becomes flatter and partially loses its saddle configuration. This dynamic of the MA is responsible for the reduction of hemodynamic stress imposed on the MV leaflets during the cardiac cycle (17). As in our study, other series reported similar results, with an increase in the MA area during systole related to the increase in the AP diameter (2,18,19).

Decloedt et al. demonstrated that delay in ring mobility results in modification of the flow dynamics and failure of coaptation of MV leaflets (20). The maintenance of MA mobility has been closely related to preservation of ventricular function and is directly proportional to left ventricular ejection fraction (2). The finding of reduced MA mobility enabled us to predict the presence of MV dysfunction and left ventricular dysfunction, with high sensitivity and specificity, and the use of flexible rings in the surgical correction did not preserve the MA dynamics (16,21). Another study demonstrated that the injury to annulus mobility was greater in techniques with greater resection of the leaflets and segmental aplications, followed by prosthetic ring annuloplasty (22). A patient-specific finite element model using CMR images has been used to individualize MVR, simulating the results of artificial chordal implantation and leaflet resection with annuloplasty, and can be useful to predict the results in ventricular and annulus dynamics (23,24).

CMR is a useful tool to detect myocardial fibrosis, which is associated with a higher incidence of arrhythmias in patients with MR and is an independent predictor of increased incidence of adverse clinical outcomes in patients undergoing MVR (25). The type and extent of myocardial fibrosis correlated with the remodeling of the left ventricle in the clinical and postoperative context of MV disease (26,27). MA abnormalities have been correlated with increased presence of fibrosis, arrhythmias, and ventricular dysfunction in studies with CMR (28), demonstrating the importance of preservation of mitral annular mobility in the treatment of MR.

## CONCLUSIONS

CMR was able to accurately demonstrate the measurements and dynamics of the MA in the pre- and postoperative periods of MVR. We observed a significant reduction in size of the MA after MVR, with preservation of MA contractility and valve repair stability at the 2 year follow-up, using a technique of MVR without ring implantation.

## AUTHOR CONTRIBUTIONS

Abdouni AA contributed in investigation, data curation, writing-original draft and writing-review & editing. Brand�o CM contributed in investigation, data curation, writing-original draft, writing-review & editing and project administration. Rochitte CE contributed in resources and data curation. Pomerantzeff PM contributed in conceptualization, investigation and supervision. Veronese ET contributed in investigation and writingreview & editing. Pacheco AB contributed in investigation. Santis AS contributed in investigation. Tarasoutchi F contributed in supervision. Jatene FB contributed in supervision and investigation.

## Figures and Tables

**Table 1:** Patients Characteristics.

Gender	N	%
Gender		
Male	17	58.6
Female	12	41.4
Preoperative Functional Class (NYHA)		
Class I	1	3.4
Class II	5	17.3
Class III	19	65.5
Class IV	4	13.8
Cardiac Rhythm on Admission		
Sinus	21	71.4
Atrial fibrillation	8	27.6
Comorbidities		
Hypertension	18	69.0
Diabetes	3	10.3
Dyslipidemia	10	34.5

Values are expressed as absolute and relative frequencies (%) for categorical values.

NYHA: New York Heart Association.

**Table 2:** Measurements of mitral annulus circumference, performed in end diastole (D) and end systole (S) and mitral valve area and antero-posterior (AP) and medial-lateral (ML) diameters of the mitral annulus, performed in diastole (D) and during the three phases of systole (S1, S2, S3).

		PRE-OP	30 DAYS	6 MONTHS	1 YEAR	2 YEARS	*p*
**Mitral Annulus Circumference (cm)**	D	12.51 (±2.01)	10.66 (±2.09)	10.88 (±1.66)	11.14 (±2.82)	10.76 (±1.61)	<0.001
	S	13.28 (±1.95)	11.50 (±1.59)	11.59 (±1.72)	11.69 (±1.99)	11.60 (±2.18)	<0.001
**Mitral Valve Area (cm^2^)**	D	12.77 (±3.72)	9.77 (±2.38)	9.65 (±2.85)	9.58 (±3.63)	9.39 (±2.81)	<0.001
	S1	12.53 (±3.68)	9.60 (±2.44)	9.66 (±2.90)	9.60 (±3.73)	9.23 (±2.84)	<0.001
	S2	13.85 (±4.05)	10.09 (±2.44)	10.70 (±3.25)	10.43 (±3.71)	10.14 (±2.94)	<0.001
	S3	14.34 (±4.03)	10.72 (±2.81)	10.92 (±3.06)	10.98 (±3.45)	10.45 (±3.17)	<0.001
**Antero-posterior Diameter (mm)**	D	38.25 (±9.79)	31.31 (±6.92)	31.01 (±7.00)	30.66 (±10.97)	30.35 (±6.44)	<0.001
	S1	37.80 (±9.41)	30.98 (±7.16)	30.79 (±6.86)	30.67 (±10.84)	30.07 (±6.40)	<0.001
	S2	40.40 (±9.84)	32.58 (±6.96)	33.58 (±7.27)	33.70 (±10.73)	32.44 (±5.88)	<0.001
	S3	42.95 (±9.98)	34.30 (±6.88)	34.54 (±6.91)	35.04 (±10.19)	33.60 (±6.75)	< 0.001
**Medial-lateral Diameter (mm)**	D	42.23 (±5.82)	39.77 (±5.52)	39.15 (±5.90)	39.57 (±5.16)	38.94 (±5.35)	=0.005
	S1	41.99 (±5.42)	39.41 (±5.40)	39.50 (±5.90)	39.54 (±5.28)	38.53 (±5.49)	=0.004
	S2	43.50 (±5.16)	39.46 (±6.24)	40.05 (±6.44)	39.28 (±5.80)	39.19 (±5.46)	<0.001
	S3	42.23 (±5.51)	39.55 (±6.31)	39.83 (±6.13)	39.81 (±5.14)	38.94 (±5.21)	=0.004

Data are expressed as mean±standard deviation; *p* values refer to comparisons between all four postoperative periods *versus* the preoperative period. D: diastole, S1: initial systole, S2: mid-systole, S3: final systole, PRE-OP: preoperative period.

**Table 3:** Measurements of variability of mitral valve area during the periods of the study.

Mitral Valve Area Variability (%)	PRE-OP	30 DAYS	6 MONTHS	1 YEAR	2 YEARS	*P*
**Minimum**	7.80	5.30	6.40	2.50	3.10	
**Maximum**	47.40	30.00	40.60	42.00	52.20	
**Mean**	23.31	19.63	23.75	25.75	22.66	0.572
**SD**	9.04	7.01	8.09	11.27	9.77	

PRE-OP: preoperative period; SD: standard deviation.
